# Statistical protein quantification and significance analysis in label-free LC-MS experiments with complex designs

**DOI:** 10.1186/1471-2105-13-S16-S6

**Published:** 2012-11-05

**Authors:** Timothy Clough, Safia Thaminy, Susanne Ragg, Ruedi Aebersold, Olga Vitek

**Affiliations:** 1 Department of Statistics, Purdue University, West Lafayette, IN, USA; 2 Department of Biology, Institute of Molecular Systems Biology, ETH Zürich, Switzerland; 3 Institute for Systems Biology, Seattle, WA, USA; 4 School of Medicine, Indiana University, Indianapolis, IN, USA; 5 Faculty of Science, University of Zürich, Switzerland; 6 Department of Computer Science, Purdue University, West Lafayette, IN, USA

**Keywords:** Label-free LC-MS/MS, linear mixed effects models, protein quantification, quantitative proteomics, statistical design of experiments

## Abstract

**Background:**

Liquid chromatography coupled with tandem mass spectrometry (LC-MS/MS) is widely used for quantitative proteomic investigations. The typical output of such studies is a list of identified and quantified peptides. The biological and clinical interest is, however, usually focused on quantitative conclusions at the protein level. Furthermore, many investigations ask complex biological questions by studying multiple interrelated experimental conditions. Therefore, there is a need in the field for generic statistical models to quantify protein levels even in complex study designs.

**Results:**

We propose a general statistical modeling approach for protein quantification in arbitrary complex experimental designs, such as time course studies, or those involving multiple experimental factors. The approach summarizes the quantitative experimental information from all the features and all the conditions that pertain to a protein. It enables both protein significance analysis between conditions, and protein quantification in individual samples or conditions. We implement the approach in an open-source R-based software package MSstats suitable for researchers with a limited statistics and programming background.

**Conclusions:**

We demonstrate, using as examples two experimental investigations with complex designs, that a simultaneous statistical modeling of all the relevant features and conditions yields a higher sensitivity of protein significance analysis and a higher accuracy of protein quantification as compared to commonly employed alternatives. The software is available at http://www.stat.purdue.edu/~ovitek/Software.html.

## Background

Liquid chromatography coupled with tandem mass spectrometry (LC-MS/MS) is widely used for relative protein quantification in complex biological mixtures. Several quantification strategies have been described that can broadly be grouped as those using stable isotope labeling and label-free methods [1-3]. Label-free methods can be further differentiated into methods based on spectral counting [4] and methods that infer analyte quantities from the ion current of the respective molecular ions. The latter method has been implemented under the term label-free LC-MS. It consists of the enzymatic digestion of proteins into a mixture of peptides, the separation of the peptides by capillary liquid chromatography, the ionization of the peptides, and the further separation of the molecular ions by the mass spectrometer according to their ratio of mass to charge. One instrument run yields a two-dimensional LC-MS profile, where peaks correspond to peptide ions, and the intensities of the peaks are related to the abundances of the peptides. The molecular ions representing specific LC-MS peaks can be further fragmented in the mass spectrometer by collision activated dissociation to generate fragment ion spectra (MS/MS), which are informative of the amino acid sequences of the peptides. The label-free shotgun LC-MS/MS workflow [5,6] is popular because it requires minimal sample processing and is relatively inexpensive. A variety of computational tools are now available to detect, quantify, and align LC-MS peaks across multiple samples and runs, and to annotate them when possible with peptide and protein identities [7].

The output of the LC-MS/MS workflow is a list of features formed by identified, quantified, and aligned LC-MS peaks. Multiple features can represent a protein when we observe differentially charged ions of the same peptide, or several peptides of the protein. In many investigations, specifically in systems biology [8] and in the discovery of biomarkers of disease [9,10], it is necessary and appropriate to *summarize all the features into protein-level conclusions*. Such summarization has two goals. First, *protein significance analysis *combines the measurements for a protein across peptides, charge states, and technical replicates across samples and conditions, and detects proteins that change in abundance between conditions more systematically than can be expected by random chance. Second, *protein quantification *combines the measurements for a protein within a particular biological sample and estimates the abundance of the protein in the sample, e.g., for use in clustering or classification. Thus, the accuracy of both protein significance analysis and protein quantification has important implications for the biological or clinical conclusions.

Many technologies other than LC-MS/MS require measurement summarization, and the solution often involves some form of averaging. E.g., in Affymetrix oligonucleotide microarrays a gene is represented by 11-16 oligonucleotides [11], and the expression of the respective gene is quantified with Tukey median polish, implemented as part of the robust multi-array averaging (RMA) normalization and summarization [12,13]. Unfortunately, a similar such summarization of the (log-)intensities of features mapped to a protein fails to produce accurate results in label-free LC-MS/MS, and it undermines the quality of the result [14]. Unlike in microarrays where the probes are designed and optimized, the LC-MS/MS workflow has little control over the observed features. It yields proteins with features that vary in number, have many interferences due to co-eluting, mis-identified or mis-quantified peptides, and have frequently missing intensities of peaks. Furthermore, the peptide ions derived from a specific protein vary in their ionization efficiency.

Measurement summarization in label-free LC-MS/MS is improved by a probabilistic modeling, which explicitly characterizes all the sources of variation. Such models have been increasingly introduced into quantitative proteomics and proven superior to averaging in both accuracy and sensitivity [14-19]. However, most of these models are applicable to restricted experiment types, e.g., the comparison of protein abundances between a limited number of conditions. To compensate for the variation and for the missing values, these models require a fairly large number of biological replicates.

In this work, we argue that the accuracy of protein significance analysis and protein quantification can be enhanced by *simultaneously studying a larger number of experimental conditions*, even when the number of replicates in each condition is relatively small. Increasing the number of conditions is advantageous biologically, because a deeper insight can be obtained by considering multiple inter-related conditions of the same model organism, or by acquiring repeated measurements on the same biological subject. It is also advantageous statistically, as it improves the accuracy of protein significance analysis and protein quantification.

In previous work, we introduced the linear mixed effects modeling framework for protein significance analysis and protein quantification in label-free LC-MS/MS experiments with simple, comparative designs [14]. The contribution of the current manuscript is to extend this work to arbitrary complex experimental designs, support the proposed framework with a software solution, and demonstrate the advantage of a joint modeling of multiple experimental conditions to practitioners with a limited statistical background. The open-source R [20]-based software package MSstats automatically recognizes the design of the experiment, and can be used for both protein significance analysis and protein quantification. We show that the framework provides meaningful results even with a moderate number of biological replicates, and in experiments with noisy and missing LC-MS peaks.

## Methods

### Dataset 1: A 3-way factorial study of breast cancer cell lines

**Figure 1:**
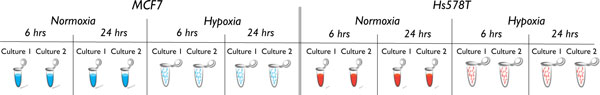
**Study of breast cancer cell lines**. Two cultures from two breast cancer cell lines (low-invasive MCF7 and high-invasive Hs578T) were treated with two oxygen levels (normoxia, hypoxia) for two periods of time (6 and 24 hours). We refer to each combination of these treatments as *condition*. Separate cultures were grown in each condition, and therefore the experiment had a 3-way factorial experimental design.

The study aimed at determining the effect of low oxygen level (hypoxia) as compared to the normal oxygen level (normoxia) on protein abundance in two breast cancer cell lines, low-invasive MCF7 and high-invasive Hs578T. Two cultures (*biological replicates*) of each cell line were treated for either 6 or 24 hours in a normoxic or hypoxic condition. The study had a complex design in that three *factors *of interest, invasive potential (low, high), treatment (hypoxia, normoxia), and time (6 h, 24 h), were examined simultaneously (Figure 1). We refer to each of the eight possible combinations of the factors as a *condition*. Since separate cultures were grown in each condition, the experiment had a *3-way factorial *design.

Independent extraction procedures [21] were performed on each culture (i.e., biological replicate), the order of the samples was randomized, and three separate mass spectrometry runs (i.e., technical replicates) were acquired for each sample on a hybrid LTQ-Orbitrap mass spectrometer (ThermoFischer Scientific, San Jose, CA, USA), producing 48 runs. LC-MS features were quantified, aligned, and annotated with peptide and protein identities using the OpenMS software [22]. The feature intensities were log-transformed and subjected to constant normalization [23]. The signal processing identified 278 proteins groups with two to 19 unambiguously mapped LC-MS features per group, and with up to 40 missing peak intensities per feature.

Additional details on the dataset are available in Supplementary Section 1.

### Dataset 2: A time course study of subjects with osteosarcoma

**Figure 2:**
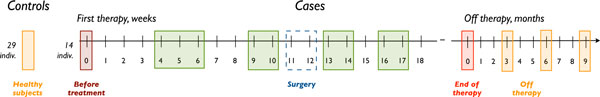
**Study of subjects with osteosarcoma**. Chemotherapy treatment was administered according to the Children's Oncology Group protocol P9754. Blood samples were collected at the weeks indicated by colored boxes before chemotherapy was administered. The study had a combination of a time course and a group comparison design.

The study was conducted at the Indiana University School of Medicine in Indianapolis. It involved 14 subjects with childhood osteosarcoma, for which serum samples were collected at multiple time points during the course of treatment, at the end of the treatment, and off-treatment (Figure 2). During the course of the study, chemotherapy and surgery were performed according to the Children's Oncology Group protocol P9754. In addition to the subjects with osteosarcoma, the study involved 29 healthy subjects for which blood samples were collected at a single time point. The institutional review board at Indiana University approved the study protocol, and written informed consent was obtained from all subjects before enrollment. The study had a complex design in that it had two *factors *of interest, disease (osteosarcoma, control) and time, and multiple measurements on the disease subjects but not on the controls. Therefore, the study had a combination of a *time course *and a *group comparison *design. Due to the difficulty of obtaining samples from subjects in longitudinal studies in a clinical setting, many subjects had missing time points.

Albumin was depleted prior to tryptic digest, the order of the samples was randomized, and each sample was analyzed in single replicate runs using Thermo-Finnigan linear ion-trap mass spectrometry, producing 130 runs. LC-MS features were quantified, aligned, and annotated with peptide and protein identities as previously described [24]. When a peak was not detected in a run the workflow reported the background noise, and therefore the dataset had no missing peak intensities. The intensities were log-transformed and subjected to quantile normalization [23]. To reduce the number of peptides with ambiguous mappings to protein isoforms, each peptide was mapped to the underlying Entrez gene ID, and the signal processing identified 121 gene groups with two to 295 peptides per group. For simplicity, we refer to the gene groups as proteins.

### Proposed significance analysis

We define protein significance analysis as a sequence of four steps: (1) *statement of the problem*, i.e., definition of the comparisons of interest and of the scope of conclusions before collecting the data, (2) *exploratory data analysis*, i.e., visualization of protein-specific features for quality control, (3) *model-based analysis*, i.e., representation of the quantitative measurements in a probabilistic model, and determination of proteins that change in abundance between conditions, while controlling the False Discovery Rate (FDR), and (4) *statistical design *of the follow-up experiments. The four steps are common to many experiments that study differences in analyte abundance, and are described, e.g., in Chang *et al. *[25]. In this manuscript we emphasize the details that are specific to label-free LC-MS/MS experiments with complex designs.

#### Step 1: Statement of the problem

Experiments with complex designs simultaneously answer multiple related questions. In the study of breast cancer cell lines we can compare, e.g., the abundance of proteins between oxygen treatments in the high invasive line, at a specific time point such as six hours of treatment. Multiple other, similar questions could be asked from the dataset. Although such comparisons can be performed by separately analyzing the runs within the respective conditions and ignoring the rest of the data, we show in Section 3 that the joint modeling of all the available conditions increases our ability to detect changes that are relevant for the specific question asked. As an illustration, here we compare the protein abundance after six hours of normoxia in the low invasive versus the high invasive cell lines, on average over the biological replicates. In the study of subjects with osteosarcoma we compare protein abundance prior to surgery (week 10) and post-surgery (week 13). The Step 3 below enables arbitrary complex comparisons of this kind.

Statement of the problem also specifies the scope of the conclusions, i.e., the interpretation that we attribute to the biological and technical replication. The *reduced scope *of biological replication means that we restrict our conclusions to the subjects in the study, e.g., to the 43 subjects in the study of osteosarcoma. This is appropriate for early-stage screening experiments. The *expanded scope *of biological replication means that we would like our conclusions to hold beyond the selected subjects, and for the underlying populations. E.g., in the study of subjects of osteosarcoma this means that we expand our conclusions beyond the selected 43 subjects, and to the entire population that these subjects represent. This is necessary for experiments at the validation stage. The choice of the scope is required for all statistical models and all experimental designs [25,26] and Step 3 below enables this choice. Section 3 illustrates that in addition to the important differences in interpretation, the scope of biological replication impacts the sensitivity and the specificity of the results. Therefore, the scope of replication should be specified according to the goals of the experiment, prior to collecting the data.

#### Step 2: Exploratory data analysis

**Figure 3:**
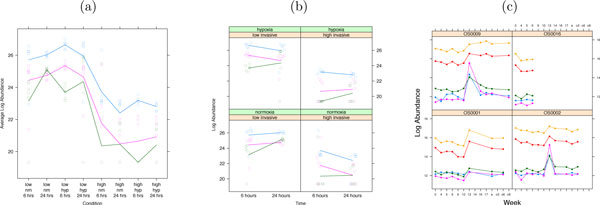
**Exploratory data analysis in **MSstats. Y-axis: Log-intensities, lines link log-intensities of LC-MS features, averaged over all replicates. (a) Quality control: profile plot of the protein SLC44A2 in the study of breast cancer cell lines. X-axis: all conditions. (b) Feature-level comparisons: trellis display of the protein SLC44A2 in the study of breast cancer cell lines. X-axis: one factor (time). Each panel: the combination of the other factors. (c) Feature-level comparisons for time course experiments: trellis display of the Entrez ID 28299 of the study of subjects with osteosarcoma. Each panel: a subject. X-axis: time.

Experiments with complex designs are more likely to induce heterogeneous stochastic variation due to the joint effects of multiple conditions on protein abundance. Therefore, MSstats implements visualization plots that assess the quality of the data and that can help to guide the downstream analysis. A *profile plot *such as in Figure 3(a) emphasizes quality control. The plot helps a user to detect mis-identified features with inconsistent quantitative profiles, or to remove features with excessive quantitative interferences or missing values. A *trellis display *[27] such as in Figure 3(b) visualizes feature-level comparisons. It helps assess whether the variation is homogenous across features and conditions, and whether changes between conditions can be due to the biological signal or to a technical artifact. Figure 3(c) is an additional trellis display for time course experiments, where each panel represents a subject. The display allows us to evaluate the consistency of both within-subject and between-subject changes in abundance.

The plots are generated automatically for all proteins in MSstats with a one-line command. The proteins selected in Figure 3 show only minor interferences in feature intensities, and the large changes between conditions appear systematic. An example of a protein with interferences is given in Supplementary Section 1.2.

#### Step 3: Model-based analysis

The conclusions regarding changes in protein abundance are formalized using statistical modeling and inference. To represent a complex experimental design we introduce a unique identifier for each *condition*, i.e., for each combination of the available experimental factors. For example, in the experiment of breast cancer cell lines each combination of cell line type, oxygen treatment, and exposure time forms one condition. In the study of subjects with osteosarcoma, each combination of disease status and time forms one condition. We further introduce a unique identifier for each *subject*, i.e., for each biological replicate.

**Figure 4:**
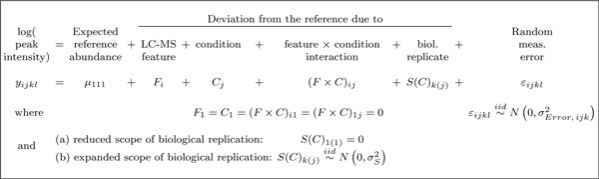
**Linear mixed effects model for a factorial experiment**. *i *= 1, ..., *I *is the index of a feature, *j *= 1, ..., *J *the index of a condition, *k *= 1, ..., *K *the index of a biological replicate, and *l *= 1, ..., *L *of a technical replicate. Notation *S*(*C*)_*k*(*j*) _is read as "biological replicate within a condition", and is the unique identifier of each biological replicate. $\sigma_{Error,ijk}^{2}$ is the variance of the measurement error and $\sigma_{S}^{2}$ the between-subject variance in the underlying population. *μ*_111 _is the expected log-intensity of the arbitrary chosen first feature, first condition, and first biological replicate. (a) and (b) are two alternative interpretations of the term *subject*, which distinguish reduced and expanded scopes of biological replication. A separate such model is specified for each protein.

The proposed linear mixed effects model for the case of factorial experiments is shown in Figure 4. The model decomposes feature intensities of a protein into the contributions of conditions (i.e., the differences in protein abundance due to the factors of interest), LC-MS features (i.e., the differences in average signal intensities between the features), and biological replicates (i.e., the natural biological differences in protein abundance between the subjects). The model accounts for interferences in the quantitative profiles of features across conditions with the statistical interaction *feature *× *condition*. For time course experiments the model is modified to account for the between-subject heterogeneity of changes in protein abundance in time (Figure 4). The model is designed for a protein quantified by at least two features, but the implementation in MSstats allows for proteins represented by a single feature by specifying a model without the *feature *and *feature *× *condition *terms.

The model expresses the reduced scope of biological replication by viewing the between-subject changes in abundance as fixed values. Alternatively, it expresses the expanded scope of biological replication by viewing these changes as randomly sampled from the underlying population. In practice a model with one of the two forms is chosen based on the desired scope of conclusions, and is specified for every protein quantified in the investigation. We show in Section 3 that the reduced scope of replication leads to higher sensitivity of detecting changes in abundance as compared to the expanded scope of replication, but that this comes at the cost of a lower specificity.

A property of label-free LC-MS/MS is that, in the absence of technical replication, biological variation is confounded with technical variation. Therefore, conclusions from the model with reduced scope of biological replication are also restricted to the performed mass spectrometry runs, unless the experiment also incorporates technical replication.

**Figure 5:**
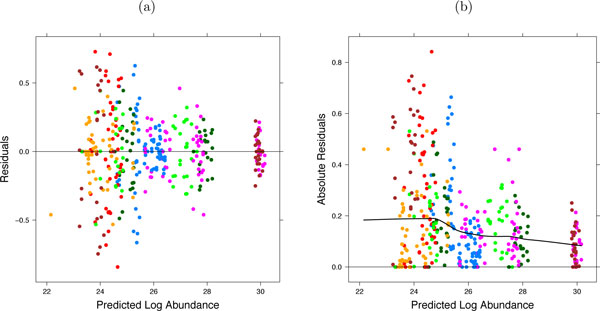
**Residual plots**. Residual plots for protein CDH13 in the study of breast cancer cell lines. The protein has 16 features, residuals from the same feature have the same color. (a) Residuals versus predicted peak log-abundance. (b) Absolute residuals versus predicted mean peak abundance are modeled by a loess curve. Values on the curve predicted for each LC-MS peak are used as weights in the iterative least squares model fit.

The technical variation in the log-intensities of LC-MS peaks is approximated relatively well by the Normal distribution. However, the extent of this variation is not necessarily constant across all features and conditions. Specifying a separate variance parameter $\sigma_{Error,ijk}^{2}$ for each feature, condition, and subject in experiments with complex designs leads to a prohibitively large number of parameters and overfits the data. Instead, MSstats implements the iterative least squares modeling procedure [28]. *Residual plots *such as in Figure 5(a) visualize the relationship between the predicted log-abundance and the unexplained technical variation. The relationship is modeled in a flexible loess fit [29] (Figure 5(b), Supplementary Section 1.3), and LC-MS peaks with larger variances have smaller weights in the resulting model-based conclusions.

**Figure 6:**
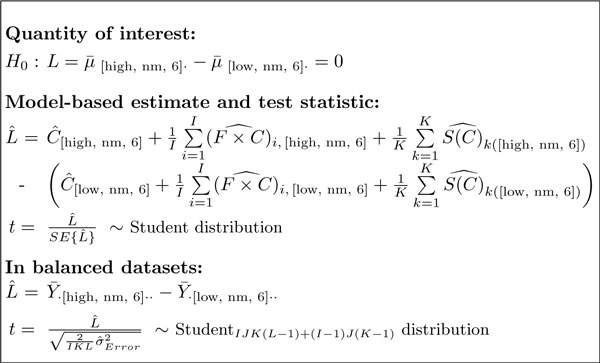
**Model-based comparison**. Model-based comparison of protein abundance between cell line types after six hours of normoxia, with reduced scope of biological replication. All notation is as in Figure 4. ${\bar{\mu }}_{\left[\text{high},\text{nm},6\right]}$. is the expected log-abundance of the protein in the high-invasive line under normoxia, after 6 hours of exposure, on average in all the observed replicates. Other conditions are denoted similarly. "^" indicates that the terms are estimated from the data.

A challenge in experiments with complex designs is in deriving the comparisons in Step 1 from this comprehensive model. In the proposed approach this is achieved by (1) translating each comparison into a model-based quantity of interest, (2) expressing the quantity as a linear combination of the model parameters, and (3) estimating this linear combination and the associated standard error from the data, separately for each protein. Figure 6 illustrates this for the factorial study of breast cancer cell lines, and Figure 5 for the study of subjects with osteosarcoma. In all investigations, a larger number of replicates provides a more accurate estimation of the quantities.

The most difficult aspect of these steps is in determining the coefficients of the linear combination. The coefficients depend on the comparison of interest, type, number, and layout of the experimental factors, on the numbers of features mapped to the protein, and on whether we reduce or expand the scope of biological replication. The implementation in MSstats automatically derives all such expressions from the input dataset and streamlines the testing for users with a limited statistics background. Finally, MSstats follows the standard testing procedure and calculates the ratio of the estimated differences and theirs standard error (in statistical terminology, the test statistic) for each protein, compares the test statistics to the Student distribution with the appropriate degrees of freedom to obtain p-values, and adjusts the p-values for multiple comparisons to control the False Discovery Rate in the list of differentially abundant proteins [30].

#### Step 4: Design of follow-up experiments

*Sample size *(i.e., the number of biological and technical replicates per condition required to detect a fold change) can be used to compare experimental strategies. In experiments with complex designs it can indicate whether the inclusion of additional conditions or time points will help detect biologically significant changes given the constraints of sample availability and cost. Sample size calculations based on linear models have been described numerous times in general [31,32] and for protein significance analysis in particular [33,34]. They take as input (1) *q*, the desired FDR in the protein list, (2) *β*, the allowed probability of not detecting a change in abundance on average over all the proteins (i.e., average Type II error), (3) *δ*, the minimal fold change in protein abundance that one would like to detect, (4) *m*_0_*/m*_0 _+ *m*_1_, the anticipated proportion of truly differentially abundant proteins in the comparison, and (5) $\sigma_{Error}^{2}$, the anticipated technical variation (and also $\sigma_{S}^{2}$, the anticipated biological variation, for models with expanded scope of biological replication). While (1)-(3) have generally accepted values, (4)-(5) depend on the specific experiment and on its biological and technical variation. MSstats derives (4) and (5) based on the current dataset, and calculates the sample size for a future investigation with a user-specified number of features, conditions, and technical replicates per condition, as described in reference [28] (see Supplementary Section 1.3.6 for illustration). We use the special case of *balanced datasets *(i.e., datasets with the same number of LC-MS runs in each condition and no missing peaks) and a representative (e.g., median) variance over all the proteins, for which the test statistic has a relatively simple form (e.g., for the study of breast cancer cell lines in Figure 6 and Supplementary Figure 3 with reduced and expanded scope of biological replication, and Supplementary Figures 5 and 6 for the time course study). Using the comparison in Figure 6 as an example, a lower bound on the fold change *δ *one can detect in a future investigation with reduced scope of biological replication is given by

(1) $$\delta^{2}\geq \frac{{\hat{\sigma }}_{\text{Error}}^{2}}{IKL}\cdot {\left(t_{1-\beta ,\;df}+t_{1-\alpha /2,\;df}\right)}^{2},\;\text{where}\;\alpha =\left(1-\beta \right)\cdot \frac{q}{1+\left(1-q\right)\cdot m_{0}/m_{1}},$$

and *t*_1-*β*, *df *_and *t*_1-*α/*2, *df *_are the 100(1 - *β*)*^th ^*and the 100(1 - *α*/2)*^th ^*percentiles, respectively, of the *t*-distribution with *df *= *IJK *(*L *- 1) + (*I *- 1) *J *(*K *- 1) degrees of freedom. From the formulae, given a fixed number of features *I*, and a fixed number of biological replicates *K *and technical replicates *L *per condition, increasing the number of related conditions *J *increases the degrees of freedom, which has the effect of decreasing the lower bound of the detectable fold change. We show this empirically in Section 3.

### Proposed protein quantification

A distinct goal of label-free LC-MS/MS investigations is *protein quantification*, i.e., the estimation of protein abundance in a biological replicate, or in one condition on average over all the replicates. In experiments with complex designs these estimates are of a particular interest, as they serve as input to machine learning tools to generate new biological knowledge from the complex datasets. For example, in the study of breast cancer cell lines we can cluster the profiles of protein abundance across conditions to find functionally related proteins. In the study of subjects with osteosarcoma the profiles can be used to predict the subject's therapy response. Such estimates of abundance differ from absolute protein quantification [35] in that they are only comparable for a same protein across conditions and runs, but not between proteins.

**Figure 7:**
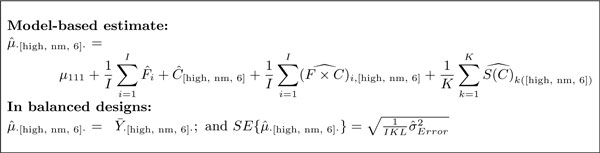
**Model-based quantification**. Model-based quantification of the expected abundance of a protein in the high invasive cell line under six hours of normoxia, on average over replicates of the condition. "^" indicates that the model-based quantities are estimated from the data.

**Figure 8:**
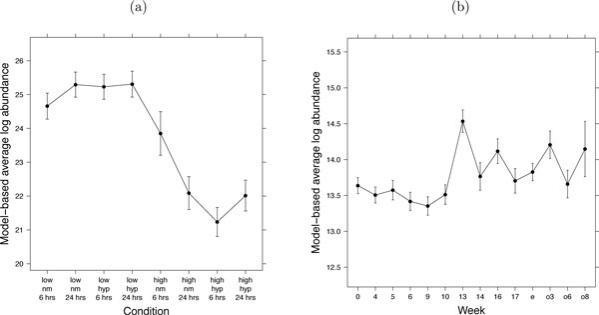
**Quantification of protein abundance in each condition**. Quantification of protein abundance in each condition, on a relative scale that is comparable between conditions of a protein but not between proteins. (a) Study of breast cancer cell lines, protein SLC44A2. (b) Study of subjects with osteosarcoma, Entrez ID 28299. X-axis: condition. Y-axis: model-based estimate of protein log-intensity. Vertical lines are the 95% confidence intervals. The selected proteins are the same as in Figure 3.

Here we argue that the accuracy of relative protein quantification is enhanced by using the same probabilistic framework as in Section 2.3. Similarly to the testing, protein abundance in a sample or condition can be expressed as a linear combination of the model parameters, and estimated from the data together with its standard error. Figure 7 illustrates the estimation of protein abundance for the condition given by six hours of normoxia treatment in the high invasive cell line in the factorial study of breast cancer cell lines. Figure 7 illustrates a similar estimation for the study of subjects with osteosarcoma. Figure 8 displays the quantifications and the associated confidence intervals for one proteins in each study, which are the same proteins as in Figure 3.

MSstats automatically derives the estimates and streamlines the estimation. In Section 3, we show that the accuracy and the precision of such protein quantification is improved when simultaneously analyzing all the available conditions.

### Proposed treatment of missing LC-MS peaks

Experiments with complex designs are particularly susceptible to missing values, as they combine intertwined effects of multiple conditions and a small sample size. An advantage of linear mixed effects models is that they tolerate missing LC-MS peaks if each feature has at least one intensity in each condition [14]. In the presence of missing data the model-based estimates of abundance differ from average log-intensities, and reflect more accurately the available information. The estimates of variation are also adjusted. E.g., the confidence intervals in Figure 8(a) vary in width due to the missing feature intensities in the study of breast cancer cell lines, and in Figure 8(b) due to missing time points in the study of subjects with osteosarcoma.

When a feature is missing entirely in one condition, this can be due to the low abundance, but also to other reasons unrelated to abundance, e.g., post-translational modifications, or even due to technical reasons. The extent of missing values is influenced by the experimental settings. There is currently no consensus on how to best account for the missing data in this case. Some authors advocate imputing missing intensities using the estimates of background noise [24,36] or by using a classification technique such as K-nearest neighbor [37]. Others argue against imputation, and transform the peak intensities to binary present/absent values [38], treat the missing intensities as censored values as in survival analysis models [18,39], or advocate testing a peptide for whether its missing intensities occur more frequently in some experimental groups than expected at random, in which case the peptide is viewed as differentially abundant [40].

Since the goal of MSstats is to flexibly represent any experimental situation, it does not enforce a one-size-fits-all treatment of missing peaks. Instead it allows the user to choose one of three options, based on prior biological expectations and on the exploratory data analysis plots. First, the user can assume that the intensities *in the specific condition *are missing due to low ion abundance, and impute the missing values in this condition with the average minimum log-intensity across runs. The analysis will reflect the interference in feature intensities, but can violate the assumptions of Normality. Second, the user can make no assumptions regarding the reason for the missing values. This will require an alternative assumption that the features have no interference (in other words, the model will not have the *feature *× *condition *statistical interaction in Figure 4). Model-based estimates will account for the missing data, but significance analysis may lose sensitivity. The third option is to assume that this is a generally poor quality feature, and remove it entirely from the dataset. In Section 3, we show that the three treatments of the missing values have only a small effect on the detection of differentially abundant proteins.

### Proposed open-source software implementation

MSstats is implemented in the open-source and open-development environment R [20]. Although the models for the factorial and the time course experiments are different, their specification in MSstats is identical. The software requires a unique identifier for each biological replicate, and uses the identities to detect the presence of repeated measurements and to internally choose the correct model.

MSstats is a series of wrappers around the generic methods lm in the library base and lmer in the library lme4 [41]. lm fits linear models where all factors are fixed, and estimates model parameters using ordinary least squares [28,42]. MSstats uses this function for models with reduced scope of biological replication. lmer fits linear mixed effects models with an arbitrary number of random effects, can handle large and unbalanced datasets, and estimates model parameters using restricted maximum likelihood [43]. MSstats uses this method for models with expanded scope of biological replication.

The use of lm and lmer is adapted to the specifics of LC-MS/MS. First, since each protein can have a different number of features, a different number of model terms (i.e., a different design matrix) is implemented for each protein. Second, options are implemented to handle heterogeneous variance and missing values. Finally, MSstats automatically derives model-based summaries for protein significance analysis and protein quantification, such that the biological comparison is the only input required from the user. Occasionally, it can be of interest to perform an analysis at the feature level instead of at the protein level, and MSstats also supports this option. Supplementary Information contains R-based code for steps of the analysis. The software and the documentation are publicly available at http://www.stat.purdue.edu/~ovitek/Software.html.

## Results

We illustrate the performance of the proposed framework in the two case studies. As an example, for the study of breast cancer cell lines, we compare protein abundances between cell line types after six hours of normoxia (i.e., the comparison in Figure 6). In all the results, for features with peak intensities missing in an entire condition the values were imputed with the average minimum log-intensity across all runs of the experiment (as implemented in MSstats) unless stated otherwise. For the study of subjects with osteosarcoma, we compare protein abundances prior to surgery (week 10) and post-surgery (week 13), as illustrated in Supplementary Section 2.2. The results for both datasets were obtained with reduced scope of biological replication unless stated otherwise.

### Result 1: Joint modeling of all conditions improves the sensitivity of protein significance analysis

**Figure 9:**
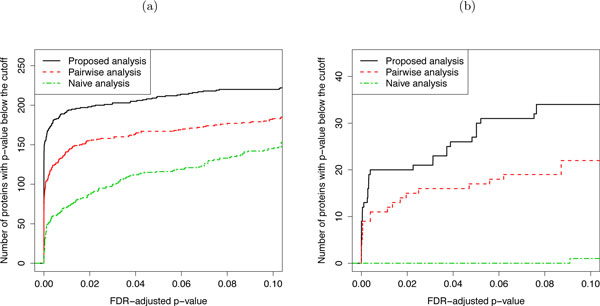
**Sensitivity of comparing protein abundance between conditions**. Sensitivity of comparing protein abundance between conditions as described at the beginning of Section 3. (a) Study of breast cancer cell lines. (b) Study of subjects with osteosarcoma. X-axis: p-values adjusted to control for the FDR using the approach by Benjamini and Hochberg. Y-axis: number of significant proteins. Solid black line: the proposed joint modeling of all conditions. Dashed red line: a linear mixed effects model that represents the conditions of interest, and ignores the rest of the data. Dashed green line: a naïve analysis, which averages feature intensities in each replicate and uses a t-test for significance analysis.

Figure 9 illustrates the sensitivity of protein significance analysis by using three approaches: (1) the *proposed analysis*, i.e., the proposed joint modeling of all conditions, (2) a *pairwise analysis*, i.e., a linear mixed effects model that only represents the conditions in the comparison, and (3) a *naïve analysis*, which averages feature intensities in each replicate, retains the averages of replicates corresponding to the conditions in the comparison, and uses a t-test for protein significance analysis. The pairwise analysis and the naïve analysis both only consider measurements from conditions in the comparison of interest. For the study of breast cancer cell lines, this amounts to ignoring all the measurements on the cell lines under the hypoxia treatment. For the study of subjects with osteosarcoma, this amounts to retaining only the measurements on subjects prior to surgery (week 10) and post-surgery (week 13), and ignoring the rest.

The figure shows, for each analysis, the number of proteins with an FDR-adjusted p-value below various p-value cut-offs. The results indicate an increased sensitivity of joint modeling over the pairwise analysis and the naïve analysis in both case studies. While one can expect, in general, a greater improvement in sensitivity by joint modeling over the alternative analyses when fewer conditions are considered in a comparison, the extent of the sensitivity gain varies between experiments, and typically increases with the sensitivity of the mass spectrometer, and decreases with technical variation in the log-intensities and with the complexity of biological samples.

### Result 2: Joint modeling of all conditions reduces the required sample size

**Figure 10:**
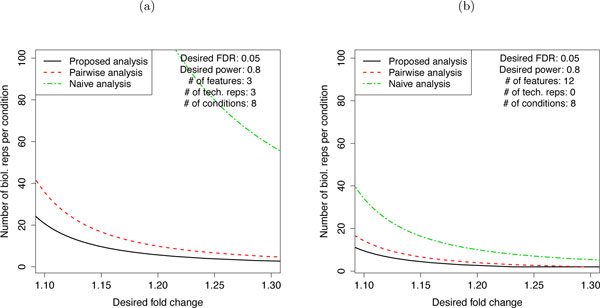
**Sample size for comparing protein abundance between conditions**. Sample size for comparing protein abundance between conditions for a representative protein, in a future experiment with the same experimental structure. (a) Study of breast cancer cell lines. (b) Study of subjects with osteosarcoma. X-axis: minimal fold change that we'd like to detect. Y-axis: the number of biological replicates per group. Solid black line: the proposed joint modeling of all conditions. Dashed red line: a linear mixed effects model that represents the conditions of interest, and ignores the rest of the data. Dashed green line: a naïve analysis, which averages feature intensities in each replicate and uses a t-test for significance analysis. The anticipated proportion of differentially abundant proteins, the number of conditions and the number of technical replicates are as in the current experiments. The number of features per protein and the error variance are set to the median values.

Figure 10 shows that a joint modeling of all conditions requires a smaller number of biological replicates to detect a given fold change than the alternative approaches, which only use measurements from the particular conditions in the comparison of interest. This is due to (1) the relationship described in Step 4 of Section 2.3, i.e., increasing the number of related conditions increases the available degrees of freedom and reduces the lower bound of the detectable standardized fold change, and (2) the fact that, although we are only interested in a subset of the conditions, the entire dataset is used to characterize the underlying variation, and yields a more accurate estimate of the variation. The difference is particularly apparent for small fold changes.

In addition to the factors outlined in Section 2.3, the sample size is strongly affected by the experimental design. A time course design, such as in the study of subjects with osteosarcoma, separates the within-subject and the between-subject variation. Therefore, a comparison of conditions (or time points) on the same subjects often leads to a smaller required sample size. This is illustrated by the smaller number of biological replicates in the time course study of osteosarcoma in Figure 10(b) as compared to the factorial design of breast cancer cell lines in Figure 10(a). As before, the extent of such differences depends on the specifics of the study.

### Result 3: Reduced scope of biological replication increases the sensitivity at the expense of specificity

**Figure 11:**
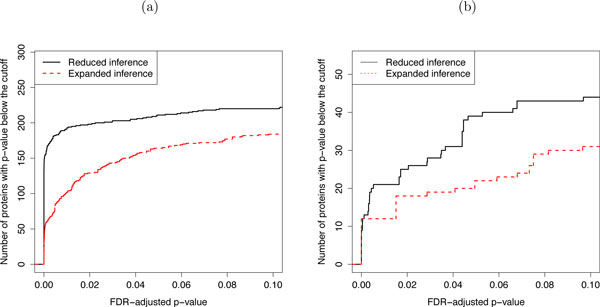
**Sensitivity of the proposed model with reduced versus expanded scope of biological replication**. Sensitivity of the proposed model with reduced versus expanded scope of biological replication for comparing protein abundances between conditions as described at the beginning of Section 3. (a) Study of breast cancer cell lines. (b) Study of subjects with osteosarcoma. X-axis: p-values adjusted to control for the FDR using the approach by Benjamini and Hochberg. Y-axis: number of significant proteins. Solid black line: reduced scope of biological replication. Dashed red line: expanded scope of biological replication.

Figure 11 illustrates the *sensitivity *of the models in both case studies. The figure shows that the reduced scope of biological replication leads to a higher sensitivity of detecting changes in abundance than the expanded scope. However, it has been previously demonstrated by simulation or in experiments with known sample composition that the sensitivity of the reduced scope of biological replication comes at the expense of lower specificity of the comparisons [14,25,44].

**Figure 12:**
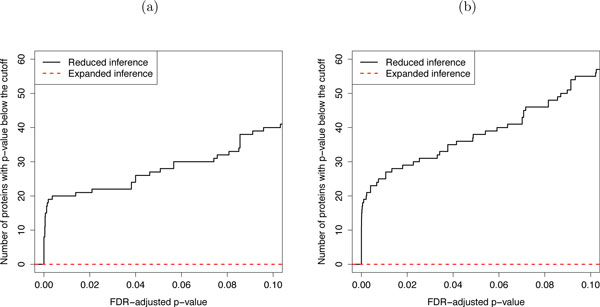
**Specificity of the proposed model with reduced versus expanded scope of biological replication**. Specificity of the proposed model with reduced versus expanded scope of biological replication in the study of breast cancer cell lines. Results from comparing protein abundances between two artificial groups of replicates within (a) the low invasive cell line under normoxia and (b) the high invasive cell line under normoxia. X-axis: p-values adjusted to control for the FDR using the approach by Benjamini and Hochberg. Y-axis: number of significant proteins. Solid black line: reduced scope of biological replication. Dashed red line: expanded scope of biological replication.

Figure 12 illustrates the *specificity *of the models in the study of breast cancer cell lines. For the purpose of illustration, for each cell line we combined the exposure time of the biological replicates under normoxia, and randomly assigned them to two artificial groups. We compared protein abundances between these two artificial groups using both reduced and expanded scope of biological replication, and recorded the number of proteins with an FDR-adjusted p-value below various p-value cut-offs. A model that identifies fewer changes has higher specificity. Figure 12 illustrates the reduction in specificity in models with reduced scope of biological replication as compared to models with expanded scope of biological replication, which successfully prohibit the discovery of any changes in both cell lines. The trade-off of sensitivity and specificity is a general and well-known property of reduced and expanded scopes of conclusions; it extends to all model types, and to all experimental designs.

### Result 4: Joint modeling of all conditions improves the precision and the accuracy of protein quantification

**Figure 13:**
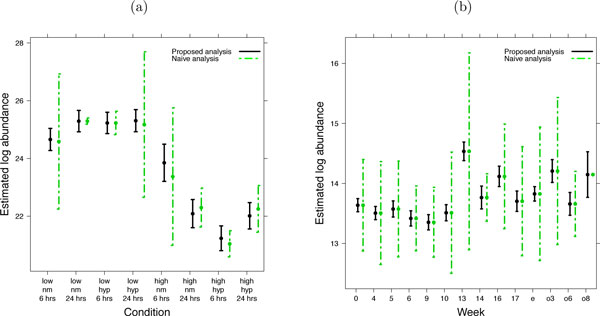
**Precision of relative protein quantifications**. (a) Study of breast cancer cell lines, protein SLC44A2. (b) Study of subjects with osteosarcoma, Entrez ID 28299. X-axis: condition. Y-axis: model-based estimate of log-abundance. Vertical lines are the 95% confidence intervals. Solid black lines: protein quantification based on the proposed joint modeling of all conditions. Dashed green lines: protein quantification based on the naïve analysis, which averages feature intensities in each replicate. The proteins are the same as in Figure 3.

Figure 13 displays protein quantifications from the proposed joint modeling of all conditions to those from the naïve analysis, which are estimated by the averages of log-intensities in each replicate. Error bars for each quantification correspond to 95% confidence intervals, reflecting the variability in the estimates. For the proposed joint modeling, the variability is quantified as shown in Figure 7. For the naïve analysis, the variability is quantified by the standard deviation of the averages. A majority of the confidence intervals from the joint analysis are narrower than those from the naïve analysis, indicating a higher precision in the quantifications.

**Figure 14:**
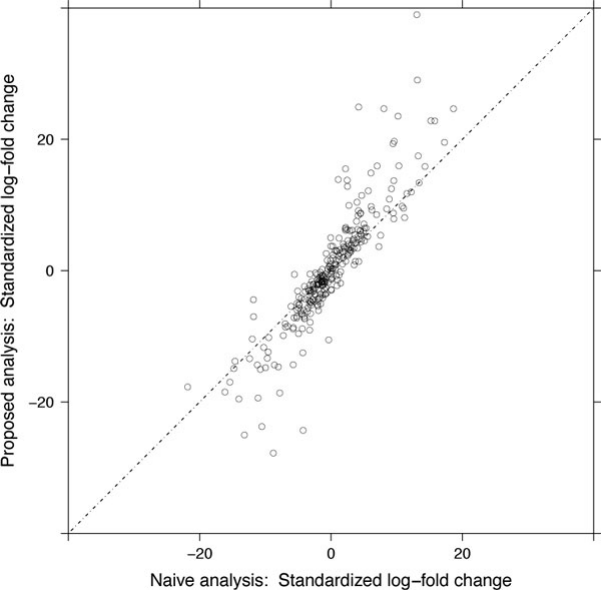
**Accuracy of protein quantifications in the study of breast cancer cell lines**. X-axis: standardized log-fold change, for the comparison described at the beginning of Section 3, from the naïve analysis, which averages feature intensities in each replicate and uses a two-group t-test. Y-axis: standardized log-fold change from the proposed joint modeling of all conditions. Dashed line: a 45° line.

For the study of breast cancer cell lines, Figure 14 further investigates the accuracy of the two approaches by comparing their standardized log-fold changes, i.e., test statistics, of the comparison of interest. The figure illustrates that the joint modeling of the LC-MS/MS intensities yields a fuller range of standardized log-fold changes, stemming from a more accurate representation of the underlying variation in the log-intensities.

### Result 5: Joint modeling of all conditions is relatively robust to the choice of treatment of missing values

**Figure 15:**
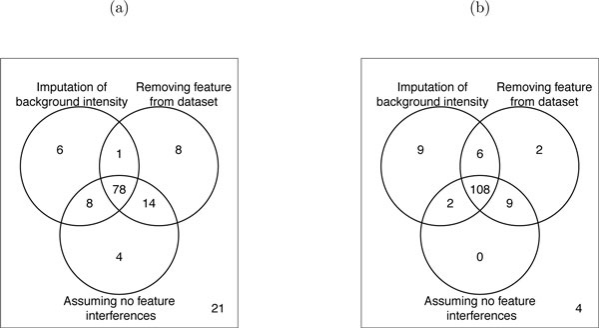
**Comparison of strategies for missing data**. Effect of the treatment of missing LC-MS peaks on protein significance analysis in the study of breast cancer cell lines, for the subset of 140 proteins where at least one feature is missing entirely in at least one condition. (a) Results of comparing protein abundances between cell line types after six hours of normoxia. (b) Results of comparing protein abundances between cell line types under normoxia, on average over the two exposure times. Each circle shows the number of differentially abundant proteins detected with three treatments of features with peak intensities missing in an entire condition: (1) imputation of the background intensity, (2) assuming no feature interferences, and (3) removing the feature from the dataset. The two comparisons have the same biological interpretation, but (b) doubles the number of biological replicates.

Due to different steps taken in data pre-processing, the dataset of breast cancer cell lines, unlike the dataset of subjects with osteosarcoma, contained numerous missing peaks. Therefore, we used this study to investigate the effect on the number of detected changes in abundance of the three treatments of proteins where at least one feature is missing entirely in at least one condition. We restricted the list of proteins in the dataset to a subset of 140 proteins with such missing values. Figure 15 shows the number of differentially abundant proteins obtained after (1) imputation of the missing condition(s) with the estimate of background intensity, (2) assuming no feature interferences, and (3) removing the feature from the dataset. Panel (a) illustrates the results for the comparison used throughout this section (low versus high invasive cell line under normoxia, with 6 hours of exposure), and panel (b) illustrates the same comparison, but on average over 6 and 24 hours of exposure times. The panels illustrate that the majority of differentially abundant proteins were identified with all three approaches. Moreover, under normoxia, the comparison in (b) has the same biological interpretation as in (a), but it doubles the number of biological replicates. As can be seen, the robustness of the conclusions to the treatment of missing values increases with the number of biological replicates.

## Discussion

Our results show that in situations where measurements are available from multiple related conditions, the proposed joint probabilistic modeling and summarization of all the LC-MS/MS runs leads to more sensitive protein significance analysis and more accurate and precise protein quantification than when separately analyzing subsets of conditions. The gain is due to a more efficient use of the data, and to a more accurate understanding of the systematic and random variation.

A distinctive feature of linear mixed effects models is the ability to distinguish two interpretations that we attribute to biological replication. The expanded scope of biological replication means that we expect to reproduce the results in a new set of biological replicates that are randomly selected from the underlying population. Significance testing based on the expanded scope is conservative, and is appropriate for confirmatory investigations. On the other hand, a model specifying the reduced scope of conclusions only expects to reproduce the results in a replicate mass spectrometry analysis of the same biological samples. Protein significance in these models may or may not be reproduced in another set of biological replicates. Such models are appropriate for small-size screening experiments. The distinction between the scopes of biological replication is a property of all biological experiments, and the advantage of linear mixed effects models is that they make this distinction explicit, and allow practitioners to make an informed choice. In all cases, the scope of conclusions should be clearly specified prior to collecting the data, the limits of generalizability should be clearly stated when reporting the results, and the conclusions should be followed by a thorough experimental validation.

Developing linear mixed effects models for experiments with complex designs and making model-based conclusions presents technical challenges for users with a limited statistics background. The implementation in MSstats automates these tasks and circumvents the challenges. Table 1 summarizes the proposed analysis workflow, Supplementary Table 2 annotates the steps of the workflow with R-based commands, and Supplementary Information contains extensive code for implementing the workflow in the two case studies. The analysis time of the workflow increases with the dimensionality of the dataset (i.e., with the number of quantified proteins, and the number of features per protein) and with the complexity of the experiment (e.g., the model-based analysis for the time course study of osteosarcoma requires a longer analysis time than the factorial study of breast cancer), but even for large-scale datasets from complex designs each step of the workflow can typically be executed within minutes.

**Table 1:** An overview of the proposed data analysis workflow.

Steps	Detailed tasks	Comments
**Statement of the problem**	• Specify comparisons of interest	• Express comparisons as statistical hypotheses
	• Define scope of biological replication	• Restricted scope suitable for screening; expanded scope required for validation

**Exploratory data analysis**	• Detect mis-identified features	• Remove obvious outliers
	• Detect features with missing values	• Choose imputation strategy

**Model-based analysis**	• Fit linear mixed model per protein	• Reduced scope of biological replication = *fixed subjects*; expanded scope = *random subjects *
	• Check qq-plots plots for Normality	• If deviations, conclusions are approximate only
	• Check residual plots for equal variance	• If deviations, use iterative least squares
	• Test comparisons of interest	• Adjust p-values per comparison to control FDR
	• Quantify protein abundance in conditions or samples of interest	• Use as input with downstream clustering or classification

**Design follow-up experiments**	• Evaluate power and sample size	• Find minimal sample size for a fold change
		• Find minimal fold change for a sample size

Supplementary Table 2 shows MSstats commands for each step.

The proposed framework, and the software implementation, should not be confused with that of the recently introduced software SRMstats for protein significance analysis in label-based selected reaction monitoring (SRM) workflows [25]. While also based on the linear mixed effects modeling framework, the class of models employed in SRMstats are designed to optimize performance in datasets specific to the SRM workflow. SRM datasets contain extra information from heavy-labeled reference peptides, which facilitates several aspects of an analysis, including normalization to remove systematic between-run variation, separation of true biological variation from non-systematic between-run variation, and a more straightforward handling of missing values within the linear mixed effects modeling framework. The framework introduced in this work is designed specifically for label-free LC-MS/MS investigations, which are characterized by a confounding of variation from multiple convoluted sources, heterogeneous stochastic variation, frequent missing data, and greater uncertainty in the conclusions. The implementation in MSstats is designed to utilize experiments with complex designs in order to better quantify the sources of variation and reduce uncertainty.

The proposed approach has several limitations, most of which are simplifications that reduce the complexity and the analysis time of large-scale datasets. First, MSstats requires the same treatment of missing peak intensities to all proteins with excessive missings. A user-specified protein-specific treatment, motivated by the quality control plots, will likely be implemented in a future version of the software. Second, MSstats applies the same class of models to all proteins in the dataset. While per-protein refinements, such as removing unnecessary interaction terms, are possible they are often impractical, slow the analysis, require adjustments for multiple testing, and may lead to more conservative tests and overfitting. Another potential problem is the assumption of Normally distributed random terms. Although this assumption is rarely satisfied exactly, in our experience the deviations from Normality on the log-intensity scale are quite minor, and linear models are known to be robust to such small deviations. Residual plots help diagnose features with major deviations, which can then be manually excluded from the analysis. The final limitation of the proposed approach is the assumption that the LC-MS features in the dataset are correctly identified, correctly mapped to protein groups, and are informative of protein abundance (e.g., are within the limits of the dynamic range). An incorrectly mapped feature, a feature close to the edge of the signal range, or a feature with a profile distorted by a post-translational modification can undermine the quality of the results. The quality control plots currently help partially alleviate this issue.

Overall, the proposed approach balances accuracy and practicality, and enables the analysis of complex experiments in high throughput. Its open-source implementation is friendly to users with a limited statistics and programming background. We hope that the proposed approach will become a valuable tool for proteomic investigations.

## Competing interests

The authors declare that they have no competing interests.

## Authors' contributions

T.C. developed the software, performed the statistical analysis, and wrote the manuscript. S.T. designed and conducted the study of breast cancer cell lines. S.R. designed and conducted the study of subjects with osteosarcoma. R.A. supervised the experimental aspects of the study of breast cancer cell lines. O.V. supervised the statistical aspects of the work and wrote the manuscript.

## Supplementary Material

Additional file 1 **Supplementary information**. Extensive experimental and computational details and R code for MSstats are provided in Supplementary information. Click here for file
