# Supplementary material for: Statistical protein quantification and significance analysis in label-free LC-MS experiments with complex designs
Source: BMC Bioinformatics. 2012 Nov 5;13(Suppl 16):S6. doi: 10.1186/1471-2105-13-S16-S6 (PMC3489535; doi:10.1186/1471-2105-13-S16-S6)
Supplement: Additional file 1 — Supplementary information. Extensive experimental and computational details and R code for MSstats are provided in Supplementary information. [file 1471-2105-13-S16-S6-S1.pdf]

Statistical protein quantification and significance analysis in label-free LC-MS experiments with complex designs

Supplementary Materials

Timothy Clough[1], Safia Thaminy[2,3], Susanne Ragg[4], Ruedi Aebersold[2,5], and Olga Vitek*[1,6]

[1]Department of Statistics, Purdue University, West Lafayette, IN, USA
[2]Department of Biology, Institute of Molecular Systems Biology, ETH Zürich, Switzerland
[3]Institute for Systems Biology, Seattle, WA, USA
[4]School of Medicine, Indiana University, Indianapolis, IN, USA
[5]Faculty of Science, University of Zürich, Switzerland
[6]Department of Computer Science, Purdue University, West Lafayette, IN, USA

Email: Olga Vitek*- ovitek@stat.purdue.edu;

*Corresponding author

1 Dataset 1: A 3-way factorial study of breast cancer cell lines

1.1 Additional experimental details on the LC-MS/MS investigation

The breast cancer cell lines MCF7 and Hs578T were purchased from ATCC (American Type Culture Collection) and cultured according to ATCC recommendations. The cell lines were incubated at 37 °C under an atmosphere of 95% air and 5% CO2. Hypoxia treatment was performed by incubating cells for 6 or 24 hours in a hypoxic chamber (Biospherix) maintained at 1% O2 and 5% CO2.

Cancer cells were harvested at confluency and scrapped using a lysis buffer (0.32M sucrose, 100 mM sodium phosphate pH 7.5 and 0.1% NP-40). The cell lysates were sonicated on ice and centrifuge at 10,000 × g for 15 minutes at 4 °C. The supernatants were carefully transferred to new tubes and protein concentration determined using the BCA protein assay kit (Pierce). An equal amount of proteins, corresponding to 30 mg of whole cell extract per cell line and treatment was used in the N-glycopeptide enrichment procedure.[1] In total, 48 samples, including biological and technical replicates, were injected randomly and analyzed using a hybrid LTQ-Orbitrap mass spectrometer (ThermoFischer Scientific, San Jose, CA, USA) interfaced with a nanoelectrospray ion source (Proxeon Biosystems). All LC-MS/MS scans were searched against the International Protein Index (IPI) human database (version 3.34) using the SEQUEST algorithm. The searching results were analyzed through the Trans Proteomic Pipeline TPP (version 4.3), including PeptideProphet and ProteinProphet. A 1% error rate at the peptide level (in this study, less than 1% FDR based on the number of decoy sequences in the remaining data set and the PeptideProphet probability score (P) $\geq$ 0.9) was applied. Quantitative analysis was performed using the open source OpenMS software.[2] Several processing steps were performed prior to statistical analysis. (i) Only MS1 features mapped to N-glycopeptides (Nx[ST] motif) and detected in at least 8 out of the 48 runs were retained. (ii) A logarithm transformation was applied to all feature intensities. (iii) A constant normalization procedure[3] was performed to remove systematic between-run variation. Overall, 1238 aligned features, corresponding to 278 unique proteins, were retained for analysis. Each N-glycoprotein contains between two to 19 features.

1.2 Exploratory data analysis

The input dataset is in "long" format, where each row corresponds to a single feature intensity, as shown in Figure 1. In what follows, the data are stored as an R structure of type `data.frame` labeled `cancer.data`, where each row represents a feature intensity in a single run and columns contain sample annotations and feature intensities.

Figure 2 is an example of exploratory data analysis in `MSstats` for protein PTPRK. Unlike the protein TMED9 in the main manuscript, this protein has features with interferences. The statistical model in Figure

A	B	C	D	E	F	G	H
	gene	peptide.charge	bio.id	Invasive	treatment	time	log2.intensity
1	KDELC2	GVTNDLLSIQGN(Deamidated)TGPSWIN(Deamidated)KTER.3	1	H	NM	24	22.4820756
2	KDELC2	GVTNDLLSIQGNTGPSWIN(Deamidated)KTER.3	1	H	NM	24	26.2047158
3	KDELC2	YFYLQAVN(Deamidated)SEGQN(Deamidated)LTR.2	1	H	NM	24	19.331757
4	KDELC2	YFYLQAVNSEGQN(Deamidated)LTR.2	1	H	NM	24	19.331757
5	KDELC2	YFYLQAVNSEGQN(Deamidated)LTR.3	1	H	NM	24	27.3191613
6	KDELC2	GVTNDLLSIQGN(Deamidated)TGPSWIN(Deamidated)KTER.3	1	H	NM	24	22.7614761
7	KDELC2	GVTNDLLSIQGNTGPSWIN(Deamidated)KTER.3	1	H	NM	24	26.3159427
8	KDELC2	YFYLQAVN(Deamidated)SEGQN(Deamidated)LTR.2	1	H	NM	24	19.331757

Figure 1: Part of the data structure used as input to MSstats in the 3-way factorial study of the breast cancer cell lines. The dataset is stored as a .csv file in "long" format.

4 in the main manuscript expresses the presence of interferences via a statistical interaction term $(F \times C)_{ij}$.

The plots as in Figure 2 can be produced separately for all proteins with a single command in MSstats, as shown below.

```
profilePlots(protein = "gene", feature = "peptide.charge", bio.rep = "bio.id",
        group = c("Invasive", "treatment", "time"), abundance = "log2.intensity",
        data = cancer.data, address = NULL,
        pointSize = 0.8, axisSize = 1, labelSize = 1, stripSize = 0.8, keySize = 0.6)
```

```
trellisPlots(protein = "gene", feature = "peptide.charge", bio.rep = "bio.id",
        group = c("Invasive", "treatment", "time"), abundance = "log2.intensity",
        data = cancer.data, address = NULL,
        pointSize = 0.8, axisSize = 1, labelSize = 1, stripSize = 0.8, keySize = 0.6)
```

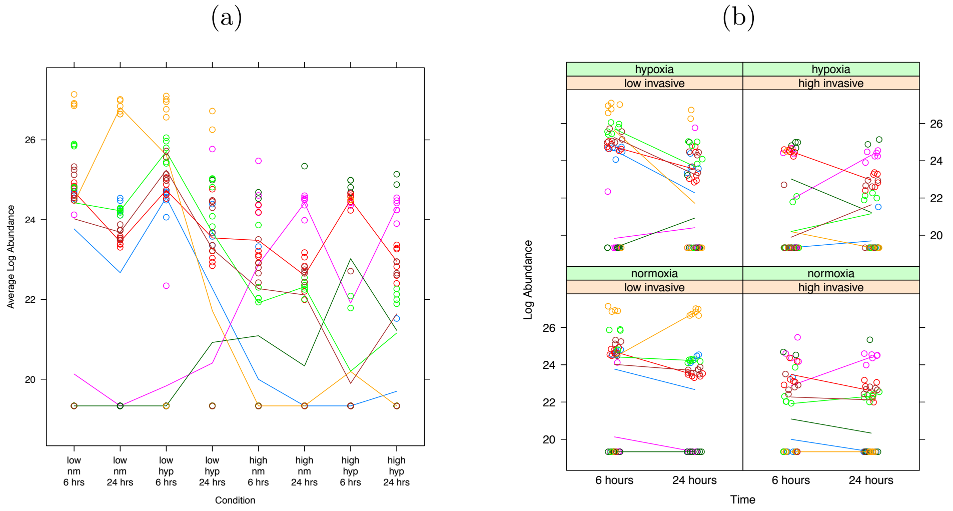

Figure 2: Exploratory data analysis in MSstats for protein PTPRK in the study of breast cancer cell lines. Y-axis: Log-intensities, lines link log-intensities of LC-MS features, averaged over all replicates. (a) Quality control. X-axis: all conditions. (b) Feature-level comparisons. X-axis: one factor (time). Each panel: a combination of the remaining factors.

1.3 Model-based analysis

In the following section we illustrate a model-based analysis in the case study of breast cancer cell lines. For features with peak intensities missing in an entire condition, we choose to impute with the average minimum log-intensity across all runs of the experiment, reflecting the strategy in the main manuscript.

1.3.1 Fit linear mixed model per protein

Reduced scope of biological replication. The model with the reduced scope of biological replication is specified separately for each protein in a dataset by entering "fixed" in the `model` argument of the `fitModels` function in `MSstats`.

```
models <- fitModels(protein = "gene", feature = "peptide.charge",
       bio.rep = "bio.id", group = c("Invasive", "treatment", "time"),
       abundance = "log2.intensity", model = "fixed",
       feature.var = FALSE, missing.action = "impute", progress = TRUE, data = cancer.data)
```

The argument `group` allows the user to consider the effect of more than a single factor on abundance. This capability is critical when multiple factors are studied. Although each name is entered separately in the `group` argument, the term *condition* in Figure 4 (main manuscript) will be created automatically for all combinations of the groups in `MSstats`.

Expanded scope of biological replication. The model with expanded scope of biological replication (Figure 4 in the main manuscript with assumption (b)) is specified for each protein in the dataset by entering "mixed" in the `model` agrument of the `fitModels` function. The specification of the remaining arguments is the same as for the models with reduced scope of biological replication.

```
models <- fitModels(protein = "gene", feature = "peptide.charge",
       bio.rep = "bio.id", group = c("Invasive", "treatment", "time"),
       abundance = "log2.intensity", model = "mixed",
       feature.var = FALSE, missing.action = "impute", progress = TRUE, data = cancer.data)
```

1.3.2 Check qq-plots for Normality

The function `qqPlots` can be used to check whether the technical variation in the log-intensities is well approximated by a Normal distribution. The function produces a normal quantile-quantile plot (qq-plot) separately for each feature mapped to a protein. If points fall approximately along a straight line for each feature, then the assumption is appropriate for that protein. Only large deviations from the line are problematic.

```
qqPlots(modelFits = models, address = NULL, pointSize = 0.8, labelSize = 1, labelSize = 1)
```

1.3.3 Check residual plots for equal variance

The model in Figure 4 in the main manuscript assumes that technical variation in the log-intensities of LC-MS peaks is well approximated by a Normal distribution with a constant variance σ^2_{Error}. Residual plots, used to visualize the heterogeneity of technical variation, can be produced for all proteins in the dataset by using the `residualPlots` function.

```
residualPlots(modelFits = models, address = NULL, pointSize = 0.8, axisSize = 1, labelSize = 1,
keySize = 0.6)
```

In practice, it is common for this variation to be highly heterogeneous. As a refinement to the model, `MSstats` can express the variances as a function of mean intensity using a loess fit[4] in a procedure called iteratively re-weighted least squares, in which intensities with large variation are given a smaller weight in the estimation of model-based quantities. This is implemented in `MSstats` with the argument `feature.var`, and it is available for models with reduced and expanded scope of biological replication.

```
models <- fitModels(protein = "gene", feature = "peptide.charge",
       bio.rep = "bio.id", group = c("Invasive", "treatment", "time"),
       abundance = "log2.intensity", model = "fixed",
       feature.var = TRUE, missing.action = "impute", progress = TRUE, data = cancer.data)
```

1.3.4 Test comparisons of interest

Here we show the steps for comparing protein abundance between various conditions in a comparison of interest. This is done using the `groupComparison` function, the input to which is an object specifying the comparison of interest, and an object containing the fitted models from `fitModels` in the previous step. For illustration we compare protein abundance between cell line types after six hours of normoxia, specified in Figure 6 in the main manuscript.

Reduced scope of biological replication. The quantities used for testing with reduced scope of biological replication are presented in Figure 6 of the main manuscript. For implementation in `MSstats`, we first express the comparison in terms of *conditions*, which can be extracted as shown. Users should create a different vector specific to their data which reflects the factors in their study.

```
conditions <- unique(paste(cancer.data$Invasive, cancer.data$treatment,
       cancer.data$time, sep = "."))
```

The command creates a vector of labels whose elements are concatenations of the three variables of interest in this study. E.g., the element `GROUPH.NM.6` represents the condition given by the high invasive cell line after six hours of normoxia.

The specific labels that correspond to the conditions in the comparison of interest are used as input to the `makeContrasts` function, which expresses the comparison in the appropriate structure for use in `groupComparison`. Here those labels are `GROUPH.NM.6` and `GROUPL.NM.6`.

```
comparison <- makeContrasts(GROUPH.NM.6 - GROUPL.NM.6, levels = conditions)
```

The `groupComparison` function simultaneously estimates the log fold change and the corresponding standard error of the estimate, and compares the ratio of the two quantities to the Student distribution with the appropriate degrees of freedom to obtain p-values.

```
results <- groupComparison(modelFits = models, contrast.matrix = comparison, progress = TRUE)
```

The p-values are adjusted to control the false discovery rate (FDR) using one of several options provided by the `topProteins` function.

```
resultsFdr <- topProteins(comparison.results = results, contrast.matrix = comparison,
       comparison.column = 1, rank.by = 1,
       number = length(results$Protein), adjust.method = "BH")
```

The output is a list of the proteins in the dataset, ranked by adjusted p-value from smallest to largest. A sample of the output is shown in Table 1 for three proteins in the dataset. It includes the estimated log-(base2) fold change and corresponding standard error, and the value of the test statistic and corresponding p-value of the comparison, separately for each protein. The log fold change is presented in the column labeled "Estimate". For protein PTPRK, the log fold change is -1.40, which corresponds to a fold change on the original scale of $-2^{1.40} = -2.64$.[1] This indicates that the protein is down-regulated in the high invasive line after six hours of normoxia, and the small p-value means that the regulation is statistically significant.

Protein	Estimate	Std. Error	t value	DF	p value	adj p value
PTPRK	-1.40	0.51	-2.77	314	0.01	0.02
KDELC2	1.29	0.56	2.31	220	0.02	0.03
TMED9	-0.66	0.36	-1.83	173	0.07	0.07

Table 1: Results of testing with reduced scope of biological replication for three proteins in the breast cancer dataset. "Estimate" is the model-based estimate of the log fold change of the comparison. P-values were adjusted using the method by Benjamini and Hochberg.

[1] The conversion will be different for different bases of logarithms, e.g., for datasets in which intensities were transformed using the natural log, the calculation will be $-e^{1.40}$.

Expanded scope of biological replication. Figure 3 presents the quantities used for testing for differential abundance in models with expanded scope of biological replication. The change in the scope of biological replication is reflected in two locations:

- The estimate of the variation of the log-fold change, given by $SE\{\hat{L}\}$, is now a function of $\hat{\sigma}^2_{Error}$ as well as $\hat{\sigma}^2_S$, the estimated variation due to the selection of biological replicates from underlying populations. The additional variance term leads to the loss of sensitivity of testing, the extent of which depends on the amount of underlying biological variation.

- The degrees of freedom of the Student distribution has decreased, additionally reflecting the expanded scope of conclusions.

The degrees of freedom of the Student distribution in Figure 3 are derived from principles of expected mean squares of variance components in an analysis of variance framework. In this work, however, quantities in proposed models are calculated based on an alternative framework known as restricted maximum likelihood (REML).[5] In models with expanded scope of biological replication based on this type of estimation, there is no generally accepted testing procedure such as the one in Figure 3. The derivation of appropriate test procedures for these models is an open area of research. As an approximation, many researchers adopt the procedure based on expected mean squares as we do here. Others argue against the use of test statistics at all, and advocate a procedure based on simulation. An advantage of the procedure we adopt here is that there exits a closed form expression of the degrees of freedom for all the models we propose, and it requires no computationally expensive simulation. The disadvantage is that the underlying framework is based on an assumption that the distribution of the test statistic is truly known, and the legitimacy of this assumption is not well-established in these models.

Despite the differences in the models and in the methodology underlying the test procedure, the implementation of the test procedure in `MSstats` for models with expanded scope of biological replication is exactly the same as for models with the reduced scope of biological replication. We reproduce the commands here.

```
conditions <- unique(paste(cancer.data$Invasive, cancer.data$treatment,
       cancer.data$time, sep = "."))
```

```
comparison <- makeContrasts(GROUPH.NM.6 - GROUPL.NM.6, levels = conditions)
```

Note that the first argument to `groupComparison` will now contain the results of the `fitModels` function with `model = "mixed"` specified, corresponding to the models with expanded scope of biological replication.

```
results <- groupComparison(modelFits = models, contrast.matrix = comparison, progress = TRUE)
```

```
resultsFdr <- topProteins(comparison.results = results, contrast.matrix = comparison,
       comparison.column = 1, rank.by = 1,
       number = length(results$Protein), adjust.method = "BH")
```

Quantity of interest:

$H_0 : L = \bar{\mu}_{\text{ [high, nm, 6]}\cdot} - \bar{\mu}_{\text{ [low, nm, 6]}\cdot} = 0$

Model-based estimate and test statistic:

$\hat{L} = \hat{C}_{\text{[high, nm, 6]}} + \frac{1}{I}\sum_{i=1}^{I}(\widehat{F \times C})_{i,\text{ [high, nm, 6]}} - \hat{C}_{\text{[low, nm, 6]}} - \frac{1}{I}\sum_{i=1}^{I}(\widehat{F \times C})_{i,\text{ [low, nm, 6]}}$

$t = \frac{\hat{L}}{SE\{\hat{L}\}} \sim$ Student distribution

In balanced designs:

$\hat{L} = \bar{Y}_{\cdot\text{[high, nm, 6]}\cdot\cdot} - \bar{Y}_{\cdot\text{[low, nm, 6]}\cdot\cdot}$

$t = \frac{\hat{L}}{\sqrt{\frac{2}{IKL}(\hat{\sigma}^2_{Error} + \hat{\sigma}^2_S)}} \sim \text{Student}_{J(K-1)}$ distribution

Figure 3: Model-based comparison of protein abundance between cell line types after six hours of normoxia, with expanded scope of biological replication. All notation is as in Figure 4 in the main manuscript. $\mu_{\text{ [high, nm, 6]}}$ is the expected log-abundance of the protein in the high-invasive line under normoxia, after 6 hours of exposure. Other conditions are denoted similarly. "$\widehat{\ }$" indicates that the terms are estimated from the data.

1.3.5 Quantify protein abundance in conditions or samples

Figure 7 in the main manuscript illustrates the estimation of protein abundance in the condition given by the high invasive cell line after six hours of normoxia. The `groupQuantification` function can be used to produce estimates for all conditions, separately for each protein. The input to the function is the output from `fitModels`, either with reduced or expanded scope of biological replication.

```
groupsQuantifications <- groupQuantification(modelFits = models, table = TRUE, progress = TRUE)
```

In a similar specification, `subjectQuantification` quantifies the abundance of the proteins in each biological replicate.

```
subjectQuantifications <- subjectQuantification(modelFits = models, table = TRUE, progress = TRUE)
```

The result of both functions is a table of the quantifications, which can easily be exported to a file.

The `adjustedMeansPlots` function produces plots of the *condition-specific quantifications*, and overlays on each quantification an error bar representing a $100(1-\alpha)\%$ confidence interval for the quantification (e.g., see Figure 8 in the main manuscript), where α is specified by the `alpha` argument in the command.

```
adjustedMeansPlots(modelFits = models, alpha = 0.05, address = NULL,
       pointSize = 1, axisSize = 1, labelSize = 1)
```

1.3.6 Design follow-up experiments

The `calculateSampleSize` function can be used to determine the number of biological replicates per condition necessary to detect a given fold change in a future label-free LC-MS/MS experiment. The function takes as input:

- `modelFits`: the fitted models from `fitModels`, with either reduced or expanded scope of biological replication. The models are used to calculate an estimate of the technical variation in the future experiment.
- `comparison`: a comparison of interest.
- `numFeatures`: a user-specified number of features in the future experiment.
- `numConditions`: a user-specified number of conditions in the future experiment.
- `numTechReps`: a user-specified number of technical replicates per condition in the future experiment.
- `diffProp`: the user-specified expected proportion of differentially abundant proteins for the comparison in the previous bullet point.
- `desiredFC`: a user-specified range of fold changes. The minimal required sample size needed to detect each fold change will be calculated by the function.
- maxn: a user-specified maximum sample size to display in the plot.
- `q`: the user-specified desired false discovery rate in the future investigation.
- `power`: the user-specified desired power of the future investigation, on average over all proteins in the investigation. Power is the probability of detecting a true change in abundance.
- `address`: location for which to store the resulting plot.

Using these quantities, the function call to produce a plot of the minimum sample size to detect each fold change in the range of fold changes is:

```
calculateSampleSize(modelFits = models, comparison = comparison,
       numFeatures = 3, numConditions = 8, numTechReps = 3, diffProp = 0.2,
       desiredFC = seq(1.1, 1.3, by = .05), q = 0.05, power = 0.8, address = NULL)
```

The arguments `numFeatures`, `numConditions`, and `numTechReps` may be left missing in the call to `calculateSampleSize`, in which case these values will be derived based on characteristics of the data in the current experiment.

1.4 Workflow for an alternative per-feature analysis

Occasionally it can be of interest to perform an analysis at the feature level instead of at the protein level. `MSstats` also supports this analysis. The workflow outlined in the previous sections remains the same, with features now treated as proteins. Specifically, the column in the input data containing feature ids should be placed in the `protein` argument of `fitModels`, and the `feature` argument should be specified by a column in the dataset given by a single value, either a number or a character. To perform the per-feature analysis in the study of breast cancer cell lines we begin by creating a variable that contains a single value in all rows.

```
cancer.data$feature <- 1
```

The call to `fitModels` will specify this column in the `feature` argument, while the actual feature ids will be entered in the `protein` argument of the function. The remaining arguments stay unchanged as shown.

```
models <- fitModels(protein = "peptide.charge", feature = "feature",
       bio.rep = "bio.id", group = c("Invasive", "treatment", "time"),
       abundance = "log2.intensity", model = "fixed",
       feature.var = FALSE, missing.action = "impute", progress = TRUE, data = cancer.data)
```

The procedure for testing for differential abundance at the feature level remains the same as in the protein-level analysis.

2 Dataset 2: A time course study of subjects with osteosarcoma

We now provide commands for an analysis of the time course study of subjects with osteosarcoma, described in Section 2.2 of the main manuscript. The data from this study are stored in an *expression set*, the second type of data format that is compatible with `MSstats`. An expression set is a structure commonly used for the storage of data from gene expression microarray experiments.

2.1 Exploratory data analysis

For the exploratory analysis, we transform the data to a data frame using the `transformData` function.

```
osteo.long <- transformData(protein = "gene_id", bio.rep = "id",
       group = c("group", "time"), data = osteoEset)
```

Since the study contains time course measurements, the `subjectSpecificPlots` function can be used to visualize subject-level variation. The function is specific to designs such as this time course, where biological replicates, i.e., subjects, are observed in multiple conditions.

```
subjectSpecificPlots(protein = "PROTEIN", feature = "FEATURE", bio.rep = "BIO.REP",
       group = "GROUP", abundance = "ABUNDANCE", data = osteo.long, address = NULL,
       pointSize = 0.8, axisSize = 1, labelSize = 1, stripSize = 0.8, keySize = 0.6)
```

In addition, the same exploratory plots as in Section 1.2 can be produced for this type of study using the following commands:

```
profilePlots(protein = "gene_id", bio.rep = "id", group = c("group", "time"),
       data = osteoEset, address = NULL,
       pointSize = 0.8, axisSize = 1, labelSize = 1, stripSize = 0.8, keySize = 0.6)
```

```
trellisPlots(protein = "gene_id", bio.rep = "id", group = c("group", "time"),
       data = osteoEset, address = NULL,
       pointSize = 0.8, axisSize = 1, labelSize = 1, stripSize = 0.8, keySize = 0.6)
```

2.2 Model-based analysis

In the following section we illustrate a model-based analysis for this case study.

2.2.1 Fit linear mixed model per protein

Reduced scope of biological replication. The model in Figure 4 with the assumption (a) in the figure specifies a model with reduced scope of biological replication for this case study. The model is different from the model used in the previous case study of breast cancer cell lines (Figure 4 in the main manuscript) in that it represents a time course experiment, and as such expresses the additional heterogeneity of changes in protein abundance between subjects in time through the $(C \times S)_{jk}$ statistical interaction term.

The following code is used to specify the model separately for each protein in the dataset:

```
models <- fitModels(protein = "gene_id", bio.rep = "id", group = c("group", "time"),
       model = "fixed", feature.var = FALSE, missing.action = "nointeraction",
       progress = TRUE, data = osteoEset)
```

Notice that the `feature` argument and the `abundance` arguments are missing from the call. This information is automatically extracted from `fitModels` when the data are stored in an expression set. When the data are not stored as an expression set, such as in the previous case study, it is required that these arguments be specified.

Notice also that the argument `missing.action = "nointeraction"` is specified. This is default treatment of proteins that have a feature which is missing entirely in one condition.[2] In this case study, however, an independent imputation step was performed prior to analysis, and so there are no missing peak intensities. As a result, the argument will have no impact on model fitting in this particular dataset.

Aside from these three arguments, the form of the call to `fitModels` is similar to the case study of breast cancer cell lines, despite the fact that the design of the two experiments is quite different. The differences in the statistical models are accounted for internally by the software, which automatically detects multiple observations on each biological replicate.

				Deviation from the reference due to										
log(peak intensity)	=	Expected reference abundance	+	peptide feature	+	condition	+	feat. × cond. interaction	+	biol. replicate	+	cond. × subj. interaction	+	Random meas. error
y_{ijkl}	=	μ_{111}	+	F_i	+	C_j	+	$(F \times C)_{ij}$	+	S_k	+	$(C \times S)_{jk}$	+	ε_{ijkl}

where $F_1 = C_1 = (F \times C)_{i1} = (F \times C)_{1j} = 0$ $\qquad \varepsilon_{ijkl} \overset{iid}{\sim} N\left(0, \sigma^2_{Error,\, ijk}\right)$

and
(a) reduced scope of biological replication: $S_1 = (C \times S)_{j1} = (C \times S)_{1k} = 0$
(b) expanded scope of biological replication: $S_k \overset{iid}{\sim} N\left(0, \sigma^2_S\right)$; $(C \times S)_{jk} \overset{iid}{\sim} N\left(0, \sigma^2_{C \times S}\right)$

Figure 4: Linear mixed effects model for a time course experiment. $i = 1, \ldots, I$ is the index of a feature, $j = 1, \ldots, J$ the index of a condition, $k = 1, \ldots, K$ the index of a biological replicate, and $l = 1, \ldots, L$ of a technical replicate. $\sigma^2_{Error,\, ijk}$ is the variance of the measurement error, σ^2_S the between-subject variance in the underlying population, and $\sigma^2_{C \times S}$ the variance due to the random interaction effects. μ_{111} is the expected log-intensity of the first feature, first condition, and first biological replicate. (a) and (b) are two alternative interpretations of the term *subject*, which distinguish reduced and expanded scopes of biological replication. A separate model is specified for each protein.

Expanded scope of biological replication. The model with expanded scope of biological replication corresponds to assumption (b) in Figure 4. In a time course experiment, expanded scope of biological replication implies that not one but two terms in the model (S_k and $(C \times S)_{jk}$) are viewed as random instances from the underlying populations. Therefore, unlike the model for expanded scope of biological replication in the case study of breast cancer cell lines, the model now contains *three* variance components: $\hat{\sigma}^2_{Error}$ and $\hat{\sigma}^2_S$ as before, and an extra term $\hat{\sigma}^2_{C \times S}$, reflecting the variation due to the random effects of the $(C \times S)_{jk}$ statistical interaction.[3]

[2]It is one of the three possible treatments of proteins with excessive missing values (described in Section 2.5 of the main manuscript).

[3]Due to the additional variance component, the model expresses two types of correlations, between peaks from the same biological replicate across conditions, and also between peaks from the same subject within a condition.[5]

The model is specified in MSstats by specifying `model = "mixed"`; the remaining arguments are the same as in the specification of the model with reduced scope of biological replication.

```
models <- fitModels(protein = "gene_id", bio.rep = "id", group = c("group", "time"),
       model = "mixed", feature.var = FALSE, progress = TRUE, data = osteoEset)
```

2.2.2 Check qq-plots for Normality and residual plots for equal variance

Code for producing qq-plots to check for Normality, and residual plots to check for equal variance, is as in Section 1.3.5.

```
qqPlots(modelFits = models, address = NULL, pointSize = 0.8, labelSize = 1, labelSize = 1)
```

```
residualPlots(modelFits = models, address = NULL, pointSize = 0.8,
       axisSize = 1, labelSize = 1, keySize = 0.6)
```

2.2.3 Test comparisons of interest

Here we show the steps for comparing protein abundances prior to surgery (week 10) and post-surgery (week 13).

Reduced scope of biological replication. The quantities used for testing the comparison using models with reduced scope of biological replication are presented in Figure 5.

The implementation of the test procedure in MSstats is the same as in the case study of breast cancer cell lines. We first frame the comparison of interest in terms of the conditions in the study, which are combinations of experimental group (osteosarcoma/control) and time. The difference in this case study lies in how we extract the labels for the conditions. Because the data are stored in an expression set, we extract the labels from the `phenoData` component as shown.

```
conditions <- unique(paste(pData(osteoEset)$group, pData(osteoEset)$time, sep = "."))
```

The labels corresponding to the conditions of interest, i.e., `GROUPOS.10`, representing osteosarcoma subjects prior to surgery (week 10), and `GROUPOS.13`, representing osteosarcoma subjects immediately post-surgery (week 13), are used to specify the comparison using `makeContrasts`.

```
comparison <- makeContrasts(GROUPOS.10 - GROUPOS.13), levels = conditions)
```

The comparison is tested, and p-values adjusted for multiple comparisons, as in the previous case study of breast cancer cell lines.

```
results <- groupComparison(modelFits = models, contrast.matrix = comparison, progress = TRUE)
```

Quantity of interest:

$H_0: L = \bar{\mu}_{[\text{disease, week:10}]\cdot} - \bar{\mu}_{[\text{disease, week:13}]\cdot} = 0$

Model-based estimate and test statistic:

$$\hat{L} = \hat{C}_{[\text{disease, week:10}]} + \frac{1}{I}\sum_{i=1}^{I}(\widehat{F \times C})_{i,[\text{disease, week:10}]} + \frac{1}{K}\sum_{k=1}^{K}(\widehat{C \times S})_{[\text{disease, week:10}],k}$$

$$- \left(\hat{C}_{[\text{disease, week:13}]} + \frac{1}{I}\sum_{i=1}^{I}(\widehat{F \times C})_{i,[\text{disease, week:13}]} + \frac{1}{K}\sum_{k=1}^{K}(\widehat{C \times S})_{[\text{disease, week:13}],k}\right)$$

$$t = \frac{\hat{L}}{SE\{\hat{L}\}} \sim \text{Student distribution}$$

In balanced designs:

$$\hat{L} = \bar{Y}_{\cdot[\text{disease, week:10}]\cdot\cdot} - \bar{Y}_{\cdot[\text{disease, week:13}]\cdot\cdot}$$

$$t = \frac{\hat{L}}{\sqrt{\frac{2}{IKL}\hat{\sigma}^2_{Error}}} \sim \text{Student}_{IJK(L-1)+(I-1)(K-1)+(I-1)(J-1)(K-1)} \text{ distribution}$$

Figure 5: Model-based comparison of protein abundances prior to surgery (week 10) and post-surgery (week 13), with reduced scope of biological replication. All notation is as in Figure 4. $\bar{\mu}_{[\text{disease, week:10}]\cdot}$ is the expected log-abundance of the protein in the osteosarcoma patients prior to surgery (week 10), on average over the biological replicates. Other conditions are denoted similarly. " $\widehat{\ }$ " indicates that the terms are estimated from the data.

```
resultsFdr <- topProteins(comparison.results = results, contrast.matrix = comparison,
        comparison.column = 1, rank.by = 1,
        number = length(osteoResults$Protein), adjust.method = "BH")
```

Expanded scope of biological replication. Figure 6 illustrates the testing procedure for models with expanded scope of biological replication. The change in the scope of biological replication is reflected in the standard error of the estimated log-fold change, which is now a function of the random error $\hat{\sigma}^2_{Error}$ and $\hat{\sigma}^2_{C\times S}$, the variance due to the random effects of the statistical interaction $C \times S$ in Figure 4.

Since this particular comparison involves two time measurements on the same subjects, the standard error dies not depend on the the estimated variance due to random effects of subjects $\hat{\sigma}^2_S$. In other comparisons, e.g., those involving comparisons of the control group to the osteosarcoma subjects, this quantity will be reflected in $SE\{\hat{L}\}$.

As in the study of breast cancer cell lines, the degrees of freedom of the Student distribution have also changed to reflect the expanded scope of biological replication. Again, we note that *the degrees of freedom are approximate* for this type of model, as they are based on principles of expected mean squares in an analysis of variance framework. As discussed in Section 1.3.4, this framework differs slightly from the framework underlying the proposed models, in which there is no consensus expression for the degrees of freedom for

expanded scope of biological replication.

Despite the differences in the model and in the testing procedure, the code in `MSstats` for estimating the quantities and testing the comparison are the same as those presented for reduced scope of replication; we reproduce those commands here.

```
conditions <- unique(paste(pData(osteoEset)$group, pData(osteoEset)$time, sep = "."))
```

```
comparison <- makeContrasts(GROUPOS.10 - GROUPOS.13), levels = conditions)
```

The first argument to `groupComparison` will now contain the results of the `fitModels` function with `model = "mixed"` specified, corresponding to the models with expanded scope of biological replication.

```
results <- groupComparison(modelFits = models, contrast.matrix = comparison, progress = TRUE)
```

```
resultsFdr <- topProteins(comparison.results = results, contrast.matrix = comparison,
       comparison.column = 1, rank.by = 1,
       number = length(osteoResults$Protein), adjust.method = "BH")
```

Quantity of interest:

$H_0 : L = \bar{\mu}_{\text{[disease, week:10]}\cdot} - \bar{\mu}_{\text{[disease, week:13]}\cdot} = 0$

Model-based estimate and test statistic:

$$\hat{L} = \hat{C}_{\text{[disease, week:10]}} + \frac{1}{I}\sum_{i=1}^{I}(\widehat{F \times C})_{i,\text{[disease, week:10]}} - \hat{C}_{\text{[disease, week:13]}} - \frac{1}{I}\sum_{i=1}^{I}(\widehat{F \times C})_{i,\text{[disease, week:13]}}$$

$$t = \frac{\hat{L}}{SE\{\hat{L}\}} \sim \text{Student distribution}$$

In balanced designs:

$$\hat{L} = \bar{Y}_{\cdot\text{[disease, week:10]}\cdot\cdot} - \bar{Y}_{\cdot\text{[disease, week:13]}\cdot\cdot}$$

$$t = \frac{\hat{L}}{\sqrt{\frac{2}{IKL}(\hat{\sigma}^2_{Error} + I \cdot \hat{\sigma}^2_{C \times S})}} \sim \text{Student}_{(J-1)(K-1)} \text{ distribution}$$

Figure 6: Model-based comparison of protein abundances prior to surgery (week 10) and post-surgery (week 13), with expanded scope of biological replication. All notation is as in Figure 4. $\bar{\mu}_{\text{[disease, week:10]}\cdot}$ is the expected log-abundance of the protein in the osteosarcoma patients prior to surgery (week 10), on average over the biological replicates. Other conditions are denoted similarly. " $\widehat{\ }$ " indicates that the terms are estimated from the data.

2.2.4 Quantify protein abundance in conditions or samples

Figure 7 illustrates the estimation of protein abundance for the group of control patients using quantities from the model in Figure 4. To produce the estimates for all conditions, separately for each protein, the `groupQuantification` can be used, with the same specification as in the case study of breast cancer cell lines. As before, the input to the function is the result of the call to `fitModels`, with either reduced or expanded scope of biological replication.

```
groupQuantifications <- groupQuantification(modelFits = models, table = TRUE, progress = TRUE)
```

In a similar specification, `subjectQuantification` quantifies the abundance of the proteins in each biological replicate.

```
subjectQuantifications <- subjectQuantification(modelFits = models, table = TRUE, progress = TRUE)
```

Figure 8(b) in the main manuscript displays the estimated proteins quantifications, and associated confidence intervals, across *conditions* for one of the proteins, Entrez ID 28299, in the dataset. Similar plots are created for all proteins using the `adjustedMeansPlots` function.

```
adjustedMeansPlots(modelFits = models, alpha = 0.05, address = NULL,
       pointSize = 1, axisSize = 1, labelSize = 1)
```

Model-based estimate:

$\hat{\mu}_{\cdot\text{[control, week:0]}\cdot} =$

$$\mu_{111} + \frac{1}{I}\sum_{i=1}^{I}\hat{F}_i + \hat{C}_{\text{[control, week:0]}} + \frac{1}{I}\sum_{i=1}^{I}\widehat{(F \times C)}_{i,\text{[control, week:0]}} + \frac{1}{K}\sum_{k=1}^{K}\hat{S}_k + \frac{1}{K}\sum_{k=1}^{K}\widehat{(C \times S)}_{\text{[control, week:0]},k}$$

In balanced designs:

$\hat{\mu}_{\cdot\text{[control, week:0]}\cdot} = \bar{Y}_{\cdot\text{[control, week:0]}\cdot}$; and $SE\{\hat{\mu}_{\cdot\text{[control, week:0]}\cdot}\} = \sqrt{\frac{1}{IKL}\hat{\sigma}^2_{Error}}$

Figure 7: Model-based quantification of the expected abundance of a protein in the control group, with reduced scope of biological replication. "$\hat{}$" indicates that the model-based quantities are estimated from the data.

2.2.5 Design follow-up experiments

As in the case study of breast cancer cell lines, the `calculateSampleSize` function produces a plot that can be used to determine the minimum number of biological replicates per condition required to detect a given fold change in a future investigation.

```
calculateSampleSize(modelFits = models, comparison = comparison,
       numFeatures = 12, numConditions = 18, numTechReps = 0, diffProp = 0.2,
       desiredFC = seq(1.1, 1.3, by = .05), q = 0.05, power = 0.8, address = NULL)
```

**Steps**	**Action to take in MSstats**
**Statement of the problem**	• Specify appropriate model for desired scope of biological replication in the `fitModels` function
**Exploratory data analysis**	• Use `trellisPlots` and `profilePlots` to detect mis-identified features or features with excessive missing values<br>• Specify strategy for treatment of missing values in `fitModels` function
**Model-based analysis**	• Use `fitModels` to fit linear mixed model per protein<br>• Use `qqPlots` to check for Normality<br>• Use `residualPlots` to check for equal variance; if deviations, specify `feature.var = TRUE` in `fitModels` and re-fit the models<br>• Use `makeContrasts`, `groupComparison`, and `topProteins` to test comparisons of interest<br>• Use `groupQuantification` and `subjectQuantification` to quantify protein abundance in conditions and samples; use `adjustedMeansPlots` to display condition-specific quantifications
**Design follow-up experiments**	• Use `calculateSampleSize` to find minimal sample size for a given fold change, or minimal fold change for a given sample size

Table 2: R-based functions for the proposed workflow in MSstats.

References

1. Zhang, H.; Li, X. J.; Martin, D.; Aebersold, R. *Nature Biotechnology* **2003,** *21,* 660–666.

2. Sturm, M.; Bertsch, A.; Gröpl, C.; Hildebrandt, A.; Hussong, R.; Lange, E.; Pfeifer, N.; Schulz-Trieglaff, O.; Zerck, A.; Reinert, K.; Kohlbacher, O. *BMC Bioinformatics* **2008,** *9,* 1–11.

3. Smyth, G. K.; Speed, T. P. *Methods* **2003,** *31,* 265–273.

4. Cleveland, W. S.; Devlin, S. J.; Grosse, E. *Journal of Econometrics* **1988,** *37,* 87 - 114.

5. Kutner, M. H.; Nachtsheim, C. J.; Netter, J.; Li, W. *Applied Linear Models;* McGraw-Hill/Irwin: New York, 5th Ed. ed.; 2005.
